# Rumen Bacteria and Serum Metabolites Predictive of Feed Efficiency Phenotypes in Beef Cattle

**DOI:** 10.1038/s41598-019-55978-y

**Published:** 2019-12-17

**Authors:** Brooke A. Clemmons, Cameron Martino, Joshua B. Powers, Shawn R. Campagna, Brynn H. Voy, Dallas R. Donohoe, James Gaffney, Mallory M. Embree, Phillip R. Myer

**Affiliations:** 1 Department of Animal Science, University of Tennessee Institute of Agriculture, Knoxville, TN USA; 2 Ascus Biosciences, Inc., San Diego, CA USA; 3 Department of Chemistry, University of Tennessee, Knoxville, TN USA; 4 Department of Nutrition, University of Tennessee, Knoxville, TN USA

**Keywords:** Applied microbiology, Microbiome

## Abstract

The rumen microbiome is critical to nutrient utilization and feed efficiency in cattle. Consequently, the objective of this study was to identify microbial and biochemical factors in Angus steers affecting divergences in feed efficiency using 16S amplicon sequencing and untargeted metabolomics. Based on calculated average residual feed intake (RFI), steers were divided into high- and low-RFI groups. Features were ranked in relation to RFI through supervised machine learning on microbial and metabolite compositions. Residual feed intake was associated with several features of the bacterial community in the rumen. Decreased bacterial α- (P = 0.03) and β- diversity (P < 0.001) was associated with Low-RFI steers. RFI was associated with several serum metabolites. Low-RFI steers had greater abundances of pantothenate (P = 0.02) based on fold change (high/low RFI). Machine learning on RFI was predictive of both rumen bacterial composition and serum metabolomic signature (AUC ≥ 0.7). Log-ratio proportions of the bacterial classes Flavobacteriia over Fusobacteriia were enriched in low-RFI steers (F = 6.8, P = 0.01). Reductions in Fusobacteriia and/or greater proportions of pantothenate-producing bacteria, such as Flavobacteriia, may result in improved nutrient utilization in low-RFI steers. Flavobacteriia and Pantothenate may potentially serve as novel biomarkers to predict or evaluate feed efficiency in Angus steers.

## Introduction

The United States is the largest producer of beef, and the beef industry accounts for a retail equivalent of $105 billion^1^. Over the next decade, demand for US exportation of beef is expected to increase^2^. Given the rapid reduction in natural resources and substantial growth of the human population expected in the coming decades, it is imperative to develop novel agricultural approaches in order to increase the global food supply with limited resources^3^. Ruminants, including beef cattle, rely on the fermentation of feedstuffs to provide energy for the animal. The rumen microbiome in cattle is fundamental for the successful conversion of plant matter to energy substrates for the animal via fermentation^4^. This microbiome also supplies the host animal with other important nutrients such as vitamins and protein^4^. Identifying and exploiting factors that affect the efficiency of this conversion in beef cattle will result in increased animal protein supply without increasing input resources.

Rumen microbes produce metabolites that are released into the rumen lumen and can be absorbed through the rumen epithelium or through the epithelium in the lower gastrointestinal tract^4^. The rumen microbes are responsible for the production of approximately 70% of the energy supply to the ruminant, including production of organic acids such as acetate and propionate^5^. Differences in the production of these metabolites, as well as variation in rate and quantity of absorption, can contribute to variation in nutrient utilization and efficiency of the ruminants, and may lead to physiological or phenotypic changes^6,7^. However, it can be difficult to distinguish the origin of many metabolites between those of endogenous origin and metabolites of microbial origin. Although associations between the rumen microbiome and physiological changes in the host have been identified^8,9^, the mechanisms driving these changes are still unknown and whether foundational, or keystone, species are responsible for the divergences in feed efficiency and other phenotypes.

In order to address these critical knowledge gaps, we used a combination of microbial genomics, metabolomics, and bioinformatics to further define variations in feed efficiency as determined by the divergence in residual feed intake (RFI). Determination of the complex associations and networks between the rumen microbiome, host metabolome, and differences in host phenotype can be facilitated by novel utilization of bioinformatics and machine learning to discover physiological patterns and microbial factors. By taking a multidisciplinary approach, research can move beyond correlation to identify variables accounting for differences in host phenotype.

The objective of this study was to identify the microbial and biochemical biomarkers mediating variation in feed efficiency in cattle. To accomplish this, we analyzed the relationships among RFI, the rumen bacterial community, and the serum metabolome.

## Results

### Sequencing information

A total number of 50 samples underwent microbial DNA extraction. Bacterial community composition was determined by amplifying and sequencing the V1-V3 hypervariable region of the 16S rRNA gene. A toal of 21,734,148 number of sequences were present following quality control and chimera removal. An average of 48,048 ± 41,628 sequences was present in each sample.

### Bacterial community diversity

**Figure 1:**
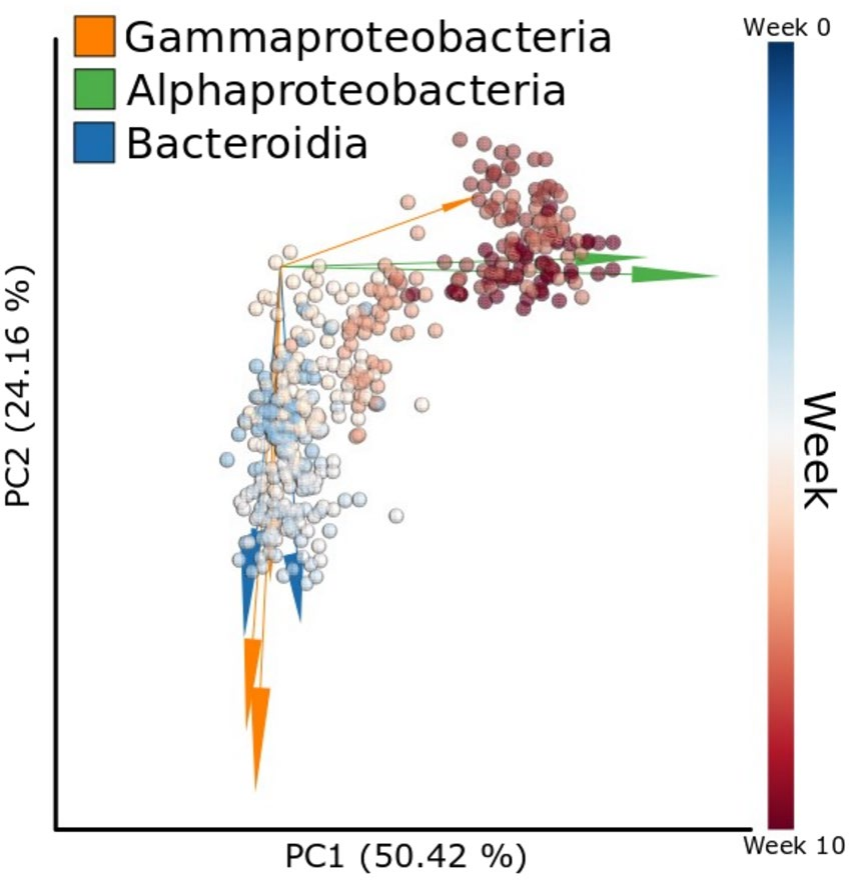
Compositional biplot beta diversity generated through Robust Aitchison PCA comparing microbial compositions over time (weeks) with arrows representing the highly ranked bacterial features colored by class level taxonomy.

After binning reads at 97% similarity, a total of 21,401 OTU were detected. Alpha-diversity was measured by equitability, Simpson’s Evenness, observed OTU, Good’s coverage, chao1, and Shannon’s Diversity Index. Alpha-diversity metrics did not differ between low- and high-RFI steers at the end of the study (Table 1), including equitability (P = 0.24; Table 1), Simpson’s Evenness (P = 0.19; Table 1), Observed OTU (P = 0.78; Table 1), Good’s coverage (P = 0.14; Table 1), chao1 (P = 0.78; Table 1), and Shannon’s Diversity Index (P = 0.07; Table 1). Beta diversity of the rumen bacterial communities also changed significantly over time (PERMANOVA: F = 422, P < 0.001, Fig. 1) with highly ranked bacterial classes Gammaproteobacteria, Alphaproteobacteria, and Bacteroidia diverging in time.

**Table 1:** Sequence and alpha-diversity statistics of the 16S rRNA gene sequences for bacterial populations in low- and high-RFI steers.

	Low-RFI^a^	High-RFI^a^	*P*-value^b^
Equitability	0.596 ± 0.040	0.650 ± 0.015	0.239
Simpson’s Evenness	0.142 ± 0.032	0.141 ± 0.011	0.190
Observed OTU	55.9 ± 4.62	59.9 ± 2.43	0.777
Good’s Coverage	0.985 ± 0.015	0.999 ± 0.001	0.140
Chao1	56.5 ± 4.27	59.9 ± 2.43	0.777
Shannon’s Diversity Index	3.38 ± 0.245	3.83 ± 0.108	0.074

^a^Mean ± SEM.

^b^Significance determined at α ≤ 0.05.

### Biochemical and microbial predictors of RFI

**Figure 2:**
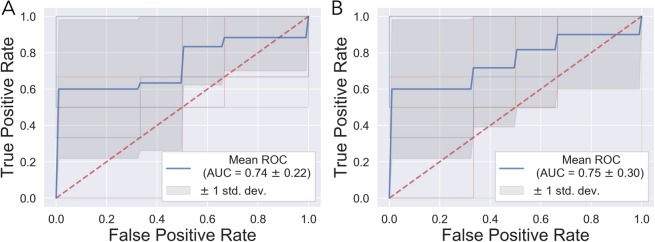
Ten-fold stratified K-Folds cross-validation ROC curves for the prediction accuracy (AUC) for bacterial (**A**) and metabolite (**B**) compositions of RFI.

**Figure 3:**
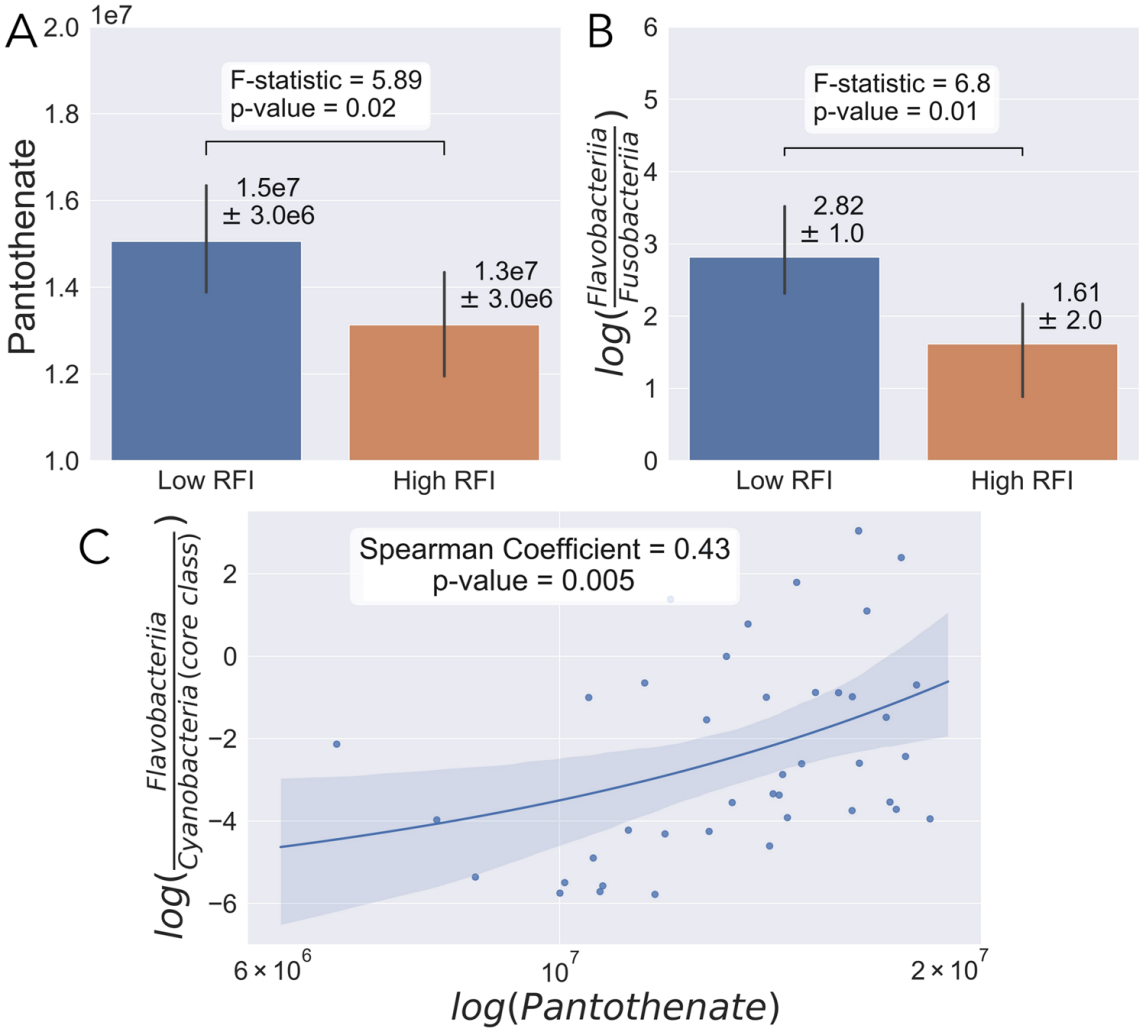
Analysis of week ten microbial compositions and metabolite abundances. Pantothenate abundance compared between low- and high-RFI (**A**). Log-ratio proportions of highly ranked microbes Flavobacteriia (numerator) and Fusobacteria (denominator) compared between low- and high-RFI (**B**). Regression plot between the log-ratio of Flavobacteriia (numerator)/Cyanobacteria (denominator) and the log-scaled pantothenate abundance.

A total of 114 known metabolites were identified. Residual feed intake was predictive of rumen bacterial composition (AUC = 0.74, Fig. 2A) and serum metabolomic composition (AUC = 0.75, Fig. 2B) at week 10 using RF machine learning and LSVC respectively. Many serum metabolites were identified as predictive of high- and low-RFI between steers, one such metabolite being pantothenate. (Supplementary Table 1). The serum pantothenate proportion was also found to be significantly different between low- and high-RFI steers (F = 5.89, P = 0.02, Fig. 3A). Additionally, among the highly ranked microbial classes were the rumen bacterial classes of Flavobacteriia and Fusobacteriia (Supplemental Table 2). The log-ratio of Flavobacteriia (numerator) and Fusobacteriia (denominator) was significantly increased in low-RFI steers (F = 6.8, P = 0.01) (Fig. 3B). Furthermore, when using Cyanobacteria, a class found at low but constant abundance across all samples as the denominator, the log ratio of Flavobacteriia (numerator) and Cyanobacteria (denominator) correlated well with pantothenate abundance (Fig. 3C).

## Discussion

Total beef consumption in the United States is greater than 20 billion pounds annually, and is the most consumed red meat product in the United States^1^; however, declining land resources and increasing human populations place pressure on producers to improve production efficiency. Therefore, researchers and producers are charged with finding novel approaches and methods for reducing inputs while increasing animal protein supply to meet the needs of an expected global population exceeding 9 billion people by the year 2050^3^. Given this need, targeting phenotypes that improve efficiencies, such a feed and reproductive efficiencies, or reduce negative environmental impacts, including methane production and excess nitrogen release, will ultimately improve beef and livestock agriculture on a global scale. The rumen microbiome contributes significantly to the breakdown of low-quality feedstuffs, such as forages, and may be responsible for much of the variation observed in ruminant feed efficiency phenotypes^10^. Understanding the relationships between host phenotypes and the rumen microbiome may provide novel methods for improving feed efficiency in ruminants.

In this study, measurements of α-diversity did not differ between the two groups. In other ecosystems, increased biodiversity is associated with greater success and resilience of the ecosystem^11,12^; however, the data presented in this study suggest that α-diversity may not be a significant contributing factor to feed efficiency phenotypes in stable bacterial communities in growing beef steers. Previous studies have also observed the same relationships between rumen bacterial α-diversity and feed efficiency phenotypes in steers^13,14^. Other factors, such as divergences in individual taxa or functionality of the rumen microbiota, may play a greater role in dictation of host feed efficiency phenotypes.

Given the relationship between feed efficiency phenotypes and the rumen bacterial communities, it may be possible to identify specific rumen microbes and serum metabolites associated with RFI. In the human vagina, for example, *Lactobacillus spp*. is often associated with pregnancy success and woman reproductive tract health^15–17^. In the rumen, specific taxa, even if at lower abundances, may cause distinct variation in feed efficiency phenotypes^18^. The lack of differences in α-diversity in previous studies suggests that divergences in feed efficiency phenotypes may be the result of dissimilarities at a finer resolution, such as individual taxa and metabolites, rather than global changes in the microbial communities and cumulative metabolites. Differences in serum and rumen metabolites may provide indications of feed efficiency and could be developed into a method for on-farm detection of feed efficiency.

The rumen microbiome produces several vital nutrients for the host animals, including organic acids that serve as glucogenic precursors, as well as proteins and vitamins^4^. A nutrient produced by the rumen microbiota is pantothenate. Pantothenate is not normally essential in the diet of adult cattle because the vitamin is produced by ruminal microbes in adequate amounts^19,20^. Pantothenate plays a significant role in the metabolism of fatty acids in ruminants and other species^21,22^. In this study, pantothenate was identified as a potential biomarker of RFI and was shown to be significantly enriched in low-RFI compared to high-RFI steers. Pantothenate is a key component of coenzyme A (CoA), which is required to perform a variety of functions in intermediary metabolism of ruminants^23^. Namely, CoA is responsible for the transfer of fatty acid components into and out of the mitochondria^24^. Pantothenate is produced by several species of bacteria in the rumen, and can then be released into the rumen lumen to be absorbed by the host animal. One class of bacteria that can generate pantothenate in the rumen are Flavobacteriia.

In this study, Flavobacteriia was also identified as a potential biomarker of RFI. However, because the absolute population of microbes in each sample is unknown, log-ratios were used to compare between low- and high-RFI^25,26^. It was found in this study that the log-ratio of Flavobacteriia and Cyanobacteria were well correlated to pantothenate abundance. Cyanobacteria was identified as a class with low-ranked and non-fluctuating proportions across all samples and used as the denominator in the log-ratio. Although, Cyanobacteria are oxygenic phototrophic bacteria they are often found at low but constant proportions in the rumen^27,28^ and thought to be possibly misclassified from the class Melainabacteria^29^. By using the log-ratio of Flavobacteriia and Cyanobacteria, the assumption can be made that this correlation is caused by an enrichment in Flavobacteriia and not a decrease in Cyanobacteria. This information supports that more efficient steers are associated with increased proportions of both Flavobacteriia and pantothenate.

In contrast to the log-ratio used in the correlation, the log-ratio of Flavobacteriia and Fusobacteria do not allow the same assumptions to be made as used above. This suggests that low-RFI animals could be caused by an increase or decrease of Flavobacteriia or an increase or decrease in Fusobacteria. *Fusobacterium necrophorum* of the class Fusobacteria is a known opportunistic pathogen and causative agent of liver abscesses in cattle^30^. Although *F. necrophorum* is a normal rumen inhabitant^31^, it is known to be enriched in high-grain diets^32^. This enrichment causes *F. necrophorum* to leak into portal circulation where it is then trapped in the liver causing abscesses^33^. Fusobacteriia and Flavobacteriia both identified as prospective important biomarkers here may play joined roles in regulating feed efficiency. However, further experimentation is needed to delineate this relationship.

Pantothenate may indicate greater feed efficiency. The relationship between pantothenate and Flavobacteriia could provide insight beyond the mechanisms accounting for some variability in feed efficiency, by possibly serving as biochemical and microbial biomarkers in the serum and rumen, respectively. These biomarkers could allow producers to identify and select animals of greater feed efficiency. Metabolites and microbes predictive of efficiency phenotypes in cattle are not only imperative to partially explaining divergences in feed efficiency, but also to the selection of microbial communities related to efficient animals. These insights may also lead to the ability to select for an optimal rumen microbiome.

This study identified potential microbial and biochemical biomarkers that were used to determine extremes in feed efficiency in steers. Although notable correlations between pantothenate and feed efficiency were identified, linking, and perhaps predicting, the functional capacity of the rumen and its microbiome, specifically Flavobacteriia, through serum pantothenate offers the potential to use serum biochemistry as an indicator in identification of feed efficient cattle. Additionally, although it has yet to be determined to what degree the rumen microbiome influences the host, or the host influences the rumen microbiome, the present study identified several key physiological elements that may impact or predict microbial community structure (e.g. RFI), or predictive of RFI (i.e. the serum metabolome and rumen bacterial community). However, it is also important to point out this is not a validation study, rather a study to initially identify potential biomarkers to inform future feed efficiency work. Future work would aim to conduct research validating these findings. As producers and researchers alike search for sources of variation in feed efficiency in cattle with the intent to optimize cattle productivity, methods to predict feed efficiency, such as use of microbial and biochemical markers could ultimately be used to improve the selection for feed efficient cattle.

## Materials and Methods

This study was approved and carried out in accordance with the recommendations of the Institutional Animal Care and Use Committee at the University of Tennessee, Knoxville.

### Animal experimental design and sample collection

Fifty weaned steers of approximately 7 months of age were housed at the Plateau Research and Education Center in Crossville, TN^34^. Animals weighed 264 ± 2.7 kg at the beginning of the study and transitioned to a backgrounding diet for 14 days prior to the start of the trial. Diet consisted of 80% corn silage, 10% cracked corn, and 10% protein supplement (11.57% crude protein and 76.93% total digestible nutrients with 28 mg monensin/kg on a dry matter basis). A 70-day feed efficiency trial was administered following the acclimation period. Steers were adapted to the GrowSafe^©^ system during that adaptation period. Body weight (BW) was measured at 7-day intervals and daily feed intake measured using the GrowSafe^©^ system for the length of the 70-day feed efficiency trial. Feed efficiency was determined using RFI^35^. At the conclusion of the trial, steers were ranked based on RFI and samples from the low- and high-RFI animals were utilized for subsequent analyses. Low- (*n* = 14) or high- (*n* = 15) RFI was determined as 0.5 SD below or above the mean RFI, respectively.

Weekly, approximately 9 mL of blood was sampled via venipuncture from the coccygeal vein into serum separator tubes (Corvac, Kendall Health Care, St. Louis, MO). Blood samples were centrifuged at 2,000 × *g* for 20 min at 4 °C. Serum was decanted into 5 mL plastic culture tubes and stored at −80 °C for further analyses. Approximately 100 mL of rumen content was collected via esophageal tubing and any content remaining on the filtered strainer was also collected^36^. Samples were transferred to 50 mL conical tubes, pH was measured using a portable pH meter, and stored at −80 °C until further processing.

### DNA extraction and amplification

Samples containing the rumen content were centrifuged for 15 min at 4,000 rpm, and the supernatant was then decanted and discarded. A volume of 0.5 mL of the remaining pellet was aliquoted for the extraction of DNA utilizing the PowerViral® Environmental RNA/DNA Isolation Kit (Mo Bio Laboratories, Inc., Carlsbad, CA, USA). The V1-V3 hypervariable regions of the bacterial 16S rRNA gene were amplified via the 27F^37^ and 534R^38^ primers modified for Illumina sequencing following the standard protocols Q5® High-Fidelity DNA Polymerase (New England Biolabs, Inc., Ipswich, MA, USA). The PCR amplicon products were then confirmed using 2% agarose gel electrophoresis. The products were purified utilizing AMPure XP beads (Beckman Coulter, Brea, CA, USA). According to standard protocols^39^, the purified libraries were subsequently quantified and sequenced on the MiSeq Platform (Illumina, San Diego, CA, USA). De-multiplexing of the raw fastq reads was performed on the MiSeq Platform (Illumina, San Diego, CA, USA).

### Phylogenetic analysis

The raw read data were phred33 quality filtered at a cutoff of 20 and adapter sequences trimmed using Trim Galore^40^. Following trimming and quality filtering, the remaining reads were filtered for low-complexity reads, cross-talk^41^, and PhiX. The 16S-based sequence clustering and taxonomic classification was executed using USEARCH UNOISE and SINTAX (v10.0.240)^42,43^ with the 16S rRNA database from RDP^44^. Each sample was filtered for sequencing depth at a minimum of 2,000 reads per sample^45^. Samples with fewer than 2,000 sequences were considered too low for adequate depth and were excluded from subsequent analyses.

### LC-MS analysis

LC-MS analysis has been described previously^34,46,47^. Briely, a volume of 50 μL from the serum samples (50 μL) of each steer was extracted for untargeted metabolomic analyses via 0.1% formic acid in acetonitrile:water:methanol (2:2:1). Separation of metabolites was completed utilizing a Synergy Hydro-RP column (100 × 2 mm, 2.5 μm particle size). The mobile phases were A: 97:3 H2O:MeOH with 11 mM tributylamine and 15 mM acetic acid and B: MeOH. The following comprised the gradient: 0.0 min, 0% B; 2.5 min 0% B; 5.0 min, 20% B; 7.5 min, 20% B; 13 min, 55% B; 15.5 min, 95% B; 18.5 min, 95% B; 19 min, 0% B, and 25 min, 0% B. A constant flow rate of 0.200 mL/min was utilized with the column temperature remaining at 25 °C. A temperature of 4 °C was maintained in the autosampler tray and a sample volume of 10 μL was injected into the Dionex UltiMate 3000 UPLC system (Thermo Fisher Scientific, Waltham, MA). To introduce the samples into an Exactive Plus Orbitrap MS (Thermo Fisher Scientific, Waltham, MA), electrospray ionization was used, conduted under an established method^47,48^.

As described previously^34,46^, the raw files were acquired from the Xcalibur MS software (Thermo Electron Corp., Waltham, MA) and ProteoWizard^49^ was used to convert data into the mzML format. The converted files were imported into the MAVEN software package (Metabolomic Analysis and Visualization Engine for LC-MS Data)^50^. Within MAVEN, the known metabolite peaks were picked, which automatically calculates peak areas across samples after implementing non-linear retention time correction. This uses a retention time window of five min and a preliminary mass error of ±20 ppm. The method of Rabinowitz and coworkers^48^ has been replicated and expanded by the UTK Biological and Small Molecule Mass Spectrometry Core (BSMMSC) and using a library of 263 retention time-accurate m/z pairs taken from MS1 spectra, final metabolite annotations were made. As part of establishing the method, the annotation parameters have been previously verified with pure standards. The metabolite mass had to be within ±5 ppm of the expected value and the eluted peak had to be found within two min of the expected retention time for a metabolite to be annotated as a known compound. Using the MAVEN software package^50^, metabolite identities were confirmed and the Quan Browser function of the Xcalibur MS Software (Thermo Electron Corp., Waltham, MA) was used for integration of peak areas for each compound.

### Statistical analyses

Beta diversity analysis was performed through Robust Aitchison PCA via deicode^51^ and the resulting biplot was visualized through EMPeror^52^. Compositional transformation of both the bacterial and metabolite data tables were performed through the centered log-ratio transform (clr)^53^ with a pseudo count of one. Feature ranking and supervised machine learning was performed on clr transformed data through Random Forests^54^. The clr transform and Permutational Multivariate Analysis of Variance (PERMANOVA) for beta diversity significance was performed through scikit-bio (http://scikit-bio.org/), data wrangling through pandas^55^, visualization through seaborn^56^ and matplotlib^57^. Random Forest Classification (RF) was performed on bacterial compositions and Linear Support Vector Classification (LSVC) was performed on metabolite compositions with default parameters through scikit-learn^58^. Additionally, ten-fold stratified K-Folds cross-validation was used to generate receiver operating characteristic (ROC) curves to evaluate the prediction accuracy under the curve (AUC) for each classification through scikit-learn.

Measurements of α-diversity, including equitability, Simpson’s Evenness E, Shannon’s Diversity Index, and Observed OTU, were assessed for normality using SAS 9.4 (SAS Institute, Cary, NC). All variables were found to follow a non-normal distribution, and were analyzed using Wilcoxon Rank Sum and Kruskal Wallis test.

## Supplementary information

Dataset 1

Dataset 2

## Data Availability

The datasets generated and/or analyzed during the current study are available from the corresponding author(s) on reasonable request.

## References

[1] ERS, U. *Cattle & Beef Statistics & Information*, 2015).

[2] USDA. USDA Long-term Projections, April (2017).

[3] Alexandratos, N. & Bruinsma, J. World agriculture towards 2030/2050: the 2012 revision. (ESA Working paper FAO, Rome, 2012).

[4] Hungate, R. E. *The rumen and its microbes*. (Elsevier, 1966).

[5] Seymour W, Campbell D, Johnson Z (2005). Relationships between rumen volatile fatty acid concentrations and milk production in dairy cows: a literature study. Animal feed science and technology.

[6] Huntington G (1990). Energy metabolism in the digestive tract and liver of cattle: influence of physiological state and nutrition. Reproduction Nutrition Development.

[7] Okine E, Mathison G (1991). Effects of feed intake on particle distribution, passage of digesta, and extent of digestion in the gastrointestinal tract of cattle. Journal of animal science.

[8] Hungate R E (1975). The Rumen Microbial Ecosystem. Annual Review of Ecology and Systematics.

[9] Fernando SC (2010). Rumen Microbial Population Dynamics during Adaptation to a High-Grain Diet. Applied and Environmental Microbiology.

[10] Bergman E (1990). Energy contributions of volatile fatty acids from the gastrointestinal tract in various species. Physiological reviews.

[11] Cardinale BJ, Palmer MA, Collins SL (2002). Species diversity enhances ecosystem functioning through interspecific facilitation. Nature.

[12] Zak DR, Holmes WE, White DC, Peacock AD, Tilman D (2003). Plant diversity, soil microbial communities, and ecosystem function: are there any links?. Ecology.

[13] McCann JC, Wiley LM, Forbes TD, Rouquette FM, Tedeschi LO (2014). Relationship between the Rumen Microbiome and Residual Feed Intake-Efficiency of Brahman Bulls Stocked on Bermudagrass Pastures. PLOS ONE.

[14] Myer PR, Smith TPL, Wells JE, Kuehn LA, Freetly HC (2015). Rumen Microbiome from Steers Differing in Feed Efficiency. PLOS ONE.

[15] Redondo-Lopez V, Cook RL, Sobel JD (1990). Emerging role of lactobacilli in the control and maintenance of the vaginal bacterial microflora. Reviews of infectious diseases.

[16] Boris S, Barbés C (2000). Role played by lactobacilli in controlling the population of vaginal pathogens. Microbes and infection.

[17] DiGiulio DB (2015). Temporal and spatial variation of the human microbiota during pregnancy. Proceedings of the National Academy of Sciences.

[18] Banerjee Samiran, Schlaeppi Klaus, van der Heijden Marcel G. A. (2018). Keystone taxa as drivers of microbiome structure and functioning. Nature Reviews Microbiology.

[19] Cole N, McLaren J, Hutcheson D (1982). Influence of preweaning and B-vitamin supplementation of the feedlot receiving diet on calves subjected to marketing and transit stress. Journal of Animal Science.

[20] Zinn R, Owens F, Stuart R, Dunbar J, Norman B (1987). B-vitamin supplementation of diets for feedlot calves. Journal of Animal Science.

[21] Smith CM, Narrow CM, Kendrick ZV, Steffen C (1987). The effect of pantothenate deficiency in mice on their metabolic response to fast and exercise. Metabolism.

[22] Palanker Musselman L, Fink JL, Baranski TJ (2016). CoA protects against the deleterious effects of caloric overload in Drosophila. Journal of Lipid Research.

[23] Ragaller V, Lebzien P, Südekum KH, Hüther L, Flachowsky G (2011). Pantothenic acid in ruminant nutrition: a review. Journal of animal physiology and animal nutrition.

[24] Ball, G. (Boca Raton: CRC Press, 2006).

[25] Morton JT (2017). Balance trees reveal microbial niche differentiation. mSystems.

[26] Gloor GB, Macklaim JM, Pawlowsky-Glahn V, Egozcue JJ (2017). Microbiome Datasets Are Compositional: And This Is Not Optional. Frontiers in microbiology.

[27] Neves ALA, Li F, Ghoshal B, McAllister T, Guan LL (2017). Enhancing the Resolution of Rumen Microbial Classification from Metatranscriptomic Data Using Kraken and Mothur. Frontiers in microbiology.

[28] Li, F. Metatranscriptomic profiling reveals linkages between the active rumen microbiome and feed efficiency in beef cattle. *Applied and environmental microbiology*, AEM. 00061–00017 (2017). 10.1128/AEM.00061-17 PMC5394315 28235871

[29] Soo RM (2014). An expanded genomic representation of the phylum cyanobacteria. Genome biology and evolution.

[30] Nagaraja TG, Chengappa MM (1998). Liver abscesses in feedlot cattle: a review. J Anim Sci.

[31] Langworth BF (1977). Fusobacterium necrophorum: its characteristics and role as an animal pathogen. Bacteriological reviews.

[32] Berg JN, Scanlan CM (1982). Studies of Fusobacterium necrophorum from bovine hepatic abscesses: biotypes, quantitation, virulence, and antibiotic susceptibility. American journal of veterinary research.

[33] Tadepalli S, Narayanan SK, Stewart GC, Chengappa MM, Nagaraja TG (2009). Fusobacterium necrophorum: a ruminal bacterium that invades liver to cause abscesses in cattle. Anaerobe.

[34] Clemmons BA (2017). Serum metabolites associated with feed efficiency in black angus steers. Metabolomics.

[35] Koch RM, Swiger LA, Chambers D, Gregory KE (1963). Efficiency of feed use in beef cattle. Journal of animal science.

[36] Paz HA, Anderson CL, Muller MJ, Kononoff PJ, Fernando SC (2016). Rumen bacterial community composition in Holstein and Jersey cows is different under same dietary condition and is not affected by sampling method. Frontiers in microbiology.

[37] Lane, D. 16S/23S rRNA sequencing. *Nucleic acid techniques in bacterial systematics* (1991).

[38] Muyzer G, De Waal EC, Uitterlinden AG (1993). Profiling of complex microbial populations by denaturing gradient gel electrophoresis analysis of polymerase chain reaction-amplified genes coding for 16S rRNA. Applied and environmental microbiology.

[39] Flores GE (2014). Temporal variability is a personalized feature of the human microbiome. Genome biology.

[40] Krueger, F. (2015).

[41] Edgar, R. C. UNCROSS: Filtering of high-frequency cross-talk in 16S amplicon reads. *bioRxiv*, 088666 (2016).

[42] Edgar, R. SINTAX: a simple non-Bayesian taxonomy classifier for 16S and ITS sequences. *BioRxiv*, 074161 (2016).

[43] Edgar RC, Flyvbjerg H (2015). Error filtering, pair assembly and error correction for next-generation sequencing reads. Bioinformatics.

[44] Cole JR (2013). Ribosomal Database Project: data and tools for high throughput rRNA analysis. Nucleic acids research.

[45] Jovel, J. *et al*. Characterization of the gut microbiome using 16S or shotgun metagenomics. *Frontiers in microbiology* **7** (2016). 10.3389/fmicb.2016.00459 PMC4837688 27148170

[46] Clemmons B (2018). Biochemical and microbial biomarkers of feed efficiency in Black Angus steers. Journal of Animal Science.

[47] Kamphorst JJ, Fan J, Lu W, White E, Rabinowitz JD (2011). Liquid chromatography–high resolution mass spectrometry analysis of fatty acid metabolism. Analytical chemistry.

[48] Lu W (2010). Metabolomic analysis via reversed-phase ion-pairing liquid chromatography coupled to a stand alone orbitrap mass spectrometer. Analytical chemistry.

[49] Chambers MC (2012). A cross-platform toolkit for mass spectrometry and proteomics. Nature biotechnology.

[50] Clasquin, M. F., Melamud, E. & Rabinowitz, J. D. LC-MS data processing with MAVEN: a metabolomic analysis and visualization engine. *Current protocols in bioinformatics*, 14.11. 11-14.11. 23 (2012). 10.1002/0471250953.bi1411s37 PMC4055029 22389014

[51] Martino C (2019). A Novel Sparse Compositional Technique Reveals Microbial Perturbations. mSystems.

[52] Vazquez-Baeza, Y., Pirrung, M., Gonzalez, A. & Knight, R. (Epub 2013/11/28, 10.1186/2047-217X-2-16 PMID: 24280061, 2013). 10.1186/2047-217X-2-16 PMC4076506 24280061

[53] Aitchison, J. Monographs on Statistics and Applied Probability (1986).

[54] Breiman L (2001). Random forests. Machine learning.

[55] McKinney, W. pandas: a foundational Python library for data analysis and statistics. *Python for High Performance and Scientific Computing*, 1–9 (2011).

[56] Waskom, M. *et al*. seaborn: v0. 7.1 (june 2016). *Zenodo*. **10** (2016).

[57] Hunter JD (2007). Matplotlib: A 2D graphics environment. Computing in science & engineering.

[58] Pedregosa F (2011). Scikit-learn: Machine learning in Python. Journal of machine learning research.

